# Extraction of Carotenoids from Pumpkin Peel and Pulp: Comparison between Innovative Green Extraction Technologies (Ultrasonic and Microwave-Assisted Extractions Using Corn Oil)

**DOI:** 10.3390/foods10040787

**Published:** 2021-04-06

**Authors:** Minaxi Sharma, Rajeev Bhat

**Affiliations:** ERA-Chair for Food (By-) Products Valorisation Technologies (VALORTECH), Estonian University of Life Sciences, Fr. R. Kreutzwaldi 1, 51006 Tartu, Estonia; rajeev.bhat@emu.ee

**Keywords:** pumpkin, carotenoids, green extraction, ultrasound-assisted extraction, microwave-assisted extraction, corn oil, antioxidant potential

## Abstract

Natural pigments improve aesthetic value as well as antioxidant potential of a food product. This study was designed to determine the effects of green extraction techniques on carotenoids, polyphenols and antioxidant activities of pulp and peel of two varieties of pumpkin (*Cucurbita maxima*). Innovative green extractions (IGE; Ultrasound and Microwave-Assisted Extractions) synergised with corn oil (used as green solvent) were compared with conventional extraction (CE; hexane/isopropyl alcohol; 60:40, *v*/*v*). Results showed total carotenoids to be almost double on employing IGE (PM2-UAE-peel = 38.03 ± 4.21; PM4-UAE-peel = 33.78 ± 1.76 µg/g) when compared to conventional extraction (PM2-CE-peel = 19.21 ± 4.39; PM4-CE-peel = 16.21 ± 2.52 µg/g). Polyphenolic contents ranged between 510.69 ± 5.50 and 588.68 ± 7.26 mg GAE/100 g of extract in IGE, compared with conventional extracts (269.50 ± 2.17 to 318.46 ± 6.60 mg GAE/100 g) and percent inhibition of 2,2-Diphenyl-1-picrylhydrazyl (DPPH) ranging between 88.32 ± 1.51 and 93.53 ± 0.30% in IGE when compared with conventional extraction (50.61 ± 1.44 to 57.79 ± 2.09%). Further, oxidative stability of carotenoids extracts from IGE (protection factor = 1.59 ± 0.01 to 1.81 ± 0.05) were found to be significantly higher (*p* < 0.05) than conventional extracts. Based on results, this study supports the use of innovative green extraction techniques to obtain bioactive pigments like carotenoids. It is anticipated that results generated will find potential applications in food, pharmaceutical and cosmetic industries.

## 1. Introduction

Recently, there has been a huge global demand for natural plant-derived pigments, which are enriched with antioxidant potentiality and can replace artificial pigments, especially in the food, pharmaceutical and cosmeceutical-based industries. Majority of the petroleum based or synthetic pigments are reported for their ill-effects on human health, which can directly induce hyperactivity and allergenicity in children and other sensitive people [1,2]. Modern day health-conscious consumers are demanding for the plant derived natural pigments, especially in food applications, which has led researches to exploit the vegetal wastes to isolate bioactive natural pigments [3]. Consumers’ demand for use of natural pigments with potential health benefits, coupled with consumer’s consciousness to acquire safe food, has rendered efforts to swap on the use of synthetic pigments with naturally derived bioactive pigments from plant sources [4]. Hence, on a global scale, food industries are copiously interested to bring in natural pigments in the food chain as a better alternative for synthetic colourants, which is expected not only to meet the consumer’s demand for safe and green food but also to meet the market challenges in relevance to regulatory policies.

Fruit and vegetable wastes and by-products obtained from food-processing industries are consists of seeds, peels and pomace. Efficient utilisation of these can bring a lot more opportunities for the extraction of several bioactive compounds including basic ingredients like polysaccharides, proteins and peptides, dietary fibres, etc., and other plant secondary metabolites such as pigments, polyphenols, antioxidants, antimicrobials, etc. [5,6], which have possible applications in food, pharma and nutraceutical industries. Among these are the natural pigments that can find potential applications as they are natural (plant-origin), safe (non-toxic), potent antioxidants, as well as can enhance the aesthetic appeal of the processed food commodities. Carotenoids, one of the major pigments, are copiously present in the food industrial wastes, and can be explored as natural colourants in food, pharma and cosmetic applications [3,7].

Pumpkin (*Cucurbita* spp.), generally used as a vegetable, belongs to Cucurbitaceae family and comprises about 27 species, mainly *Cucurbita maxima*, *Cucurbita pepo* and *Cucurbita moschata* [8]. Besides different species of pumpkins, there are also numerous varieties, which differ in shape, colour and chemical composition on the basis of geographical conditions of the same species. Pumpkins are rich source of carotenoids, which are precursor of vitamin A, a major antioxidant and a good natural colouring compound that is present mainly in pulp and peels, along with other bioactive compounds such as polyphenolic compounds, minerals and vitamin C [9]. A plethora of epidemiological studies reported that high intake of carotenoids in diet are related with lower risks of chronic diseases such as cardio-vascular diseases (CVDs), cancer, neurological disorder or eye-related diseases [10]. Carotenoids being potent antioxidant have been used as colouring compounds or natural dye in many food applications such as frozen desserts, salad dressings, butter, roasted foods (popcorn), in some beverages, etc. [11,12]. The growing consumer demand for natural pigments with antioxidant potential presents an opportunity for utilisation of pumpkin wastes for the exploitation of pigments for food industries. In this regard, pumpkin could be a great source of natural colouring as well as antioxidant compounds, attributing to its rich carotenoids content, having potential applications in food formulations.

In the 21st century, researchers are more concerned about consumers’ health and environment, and in the meantime are aiming towards enhancing competition of academia and industries to make them more ecologic, economic and innovative [13]. Green extraction technologies for valorisation of food wastes and by-products are safe for human health and support green consumerism; are natural, promoting the use of plant-bioactive compounds; and are eco-friendly, reducing the energy consumption and eradicating the petro-based chemicals (organic solvents) from the foods, which ultimately supports the green and circular economy.

In this framework, utilisation of vegetable oils as green solvent is gaining huge advantages in the area of natural pigments extraction with their potent antioxidant activities from food waste to maintain green label on the food formulations as well as stabilise the pigment in oil medium. Several studies investigated the potentiality of using vegetable oils as a green solvent/co-solvent along with other innovative technologies for the extraction of carotenoids from fruits and vegetables wastes/their by-products such as mandarin epicarp using sunflower oil [14], carrots using canola oil [15], tomato by utilising almond oil, sunflower seed oil, peanut oil and hazelnut oil [16], carrot using sunflower oil [17], etc. However, none of the studies have reported on the extraction of carotenoids from pumpkin waste (pulp and peels) using green solvents (especially corn oil).

In the present study, to support green consumerism, innovative extraction techniques such as ultrasound and microwave-assisted extractions were employed along with green solvent (corn oil) to valorise carotenoids from pumpkin (pulp and peels) to harvest their synergistic effects as a natural colourant with antioxidant capacity. The findings are expected to have applications in various industrial sectors such as food, pharmaceutical and cosmetics.

## 2. Materials and Methods

### 2.1. Chemicals and Reagents

Folin-Ciocalteau reagent, gallic acid, 6-hydroxy 2,5,7,8-tetramethyl-chroman-2-carboxylic acid (Trolox), 2,2-Diphenyl-1-picrylhydrazyl (DPPH) were procured from Sigma-Aldrich. Sodium carbonate was procured from Fisher Scientific (Leicestershire, UK). The extraction solvents hexane and isopropyl alcohol (isopropanol) were purchased from Merck (Darmstadt, Germany). Corn oil (refined) was purchased from local market of Tartu city (RIMI, Eesti Food AS, Pildiküla, Estonia). All chemicals, solvents (methanol and ethanol) and reagents used were of analytical grade.

### 2.2. Sample Preparation

Pumpkin species *Cucurbita maxima* with two varieties Gold Nugget (code: PM2) and Amoro F1 (code: PM4) were purchased from local market in Tartu city and coded as PM2 and PM4. Pulp and peels of these pumpkins were separated manually and chopped into small pieces. Both the pulp and peels were dried using freeze dryer followed by grinding in a laboratory grinder. The powdered samples were packed in plastic zipper bags via vacuum packaging and immediately kept at −20 °C, to protect from oxygen and light prior to extraction.

### 2.3. Extraction Methods for Carotenoids from Pumpkin Peels and Pulp

**Figure 1:**
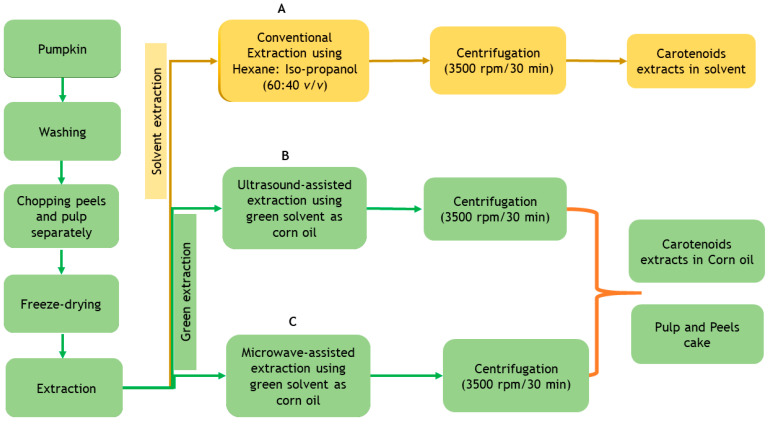
Process for the production of carotenoid extracts using different extraction methods (**A**) conventional extraction (CE) using organic solvents, (**B**) ultrasound-assisted extraction (UAE) using corn oil as green solvent, and (**C**) microwave-assisted extraction (MAE) using corn oil as green solvent.

Figure 1 represents the integrated process employed for the preparation of pumpkin peel and pulp freeze-dried powders and different extraction treatments such as conventional (CE), ultrasound-assisted (UAE) and microwave-assisted extractions (MAE) for the extraction of carotenoids as natural pigments. Pulp and peels of both the varieties of pumpkin were treated with different extraction treatments (UAE, MAE and CE) and the effect of different extraction techniques on the carotenoids content, physico-chemical and antioxidant activities were analysed. Figure 1 represents the comparison between conventional extraction (using organic solvents—hexane and isopropyl alcohol) and green extraction (UAE and MAE using corn oil as alternative solvent) techniques. Table 1 presents the experimental design for a comparative analysis of total carotenoids content and other bioactive potential of pumpkin extracts by using different extraction techniques (UAE, MAE and CE) with processing variables.

#### 2.3.1. Conventional Solvent Extraction (CE)

For the conventional extraction (CE) of carotenoids, we followed the method proposed by Goula et al. [18] with some modifications. For extraction, 5 g of pumpkin peel and pulp samples (freeze-dried) were accurately weighed and 25 mL of solvent mixture of hexane/isopropyl alcohol (60:40 *v*/*v*) was added in each sample for extraction of carotenoids. The extraction was repeated four times until complete extraction of carotenoids (no visible yellow colour) from sample. In order to achieve the phase separation and to eliminate traces of isopropanol at each of the progressive steps, the extracts were washed with equal volume of 0.1% NaCl solution. The extracts were placed in hot air oven (45 °C) to evaporate the solvent (up to 50 mL) to make equal volume as in green extractions (corn oil, see Section 2.3.2). The carotenoids extracts were collected and stored at −20 °C for further estimation of total carotenoids. As the pigments are light sensitive, all the investigations were performed in dark.

#### 2.3.2. Ultrasound-Assisted Extraction (UAE) Using Corn Oil (CO) as Green Solvent

To facilitate the green-extraction of carotenoids, ultrasound-assisted extraction (UAE) method was utilised as proposed by Goula et al. [18], after some modifications. To achieve UAE extraction (Digital Sonifier^®^ S450 CE, Branson Ultrasonics Co., Danburry, CT, USA) of carotenoids, 5 g of pumpkin peel and pulp samples were weighed in extraction flask followed by addition of 50 mL of corn oil (1:10, sample/oil). The ultrasonic probe of 13 mm (Digital Sonifier^®^ S450 CE, Branson Ultrasonics Co., Danburry, CT, USA) was immersed in the sample (peel/pulp powder and corn oil) at an amplitude of 20% for 30 min (actual time 30 min; with on/off 45 min). The pulse duration was adjusted to “on” (10 s) and “off” (5 s) mode during extraction process. Rise in temperature was manipulated about 22–25 °C via condensation accessories (using running tap water) with UAE instrument. Corn oil was used as alternative green solvent for extraction. The extracts were collected after extraction and centrifuged (Sigma Laborzentrifugen GmbH, 3-18KS, Osterode am Harz, Germany) at 4500 rpm for 45 min for complete separation of oil and residue, and later on stored at −20 °C for further analysis.

#### 2.3.3. Microwave-Assisted Extraction (MAE) Using Corn Oil (CO) as Green Solvent

For the second green-extraction technique of carotenoids, microwave-assisted extraction (MAE) method was followed according to Chuyen et al. [19] with modifications. Briefly, 5g of pumpkin peel and pulp samples were weighed in extraction flask followed by addition of 50 mL of corn oil (1:10, sample/oil) and kept into MAE extractor (NEOS-GR, Microwave gravity station, Milestone, SK, Canada) chamber adjusted with a specific clamp stand. The condition setup for the experiment was 130 W for 30 min. To maintain the temperature (max. 45 °C), the flask was manually removed from the extraction chamber and kept in ice-bath (after each 5 min) to cool down, to avoid unnecessary rise in temperature. The extracts were collected after extraction and centrifuged (Sigma Laborzentrifugen GmbH, 3-18KS, Osterode am Harz, Germany) at 4500 rpm for 45 min for complete separation of oil and residue, and then stored for further analysis at −20 °C.

### 2.4. Determination of Total Carotenoids Content (TCC)

The total carotenoids content of corn oil-carotenoids extracts from pumpkins was measured by the method suggested by Goula et al. [18], with minor modifications. For the estimation of TCC, 3 g of the extracts were accurately weighed in falcon tubes and cyclohexane was added to make the total volume up to 10 mL. These oil solutions were assessed for absorbance value (A) by using spectrophotometer (SPECORD-250 Plus, Analytik Zena, Germany) at 470 nm and the total carotenoids content was calculated by taking specific coefficient for lutein, Eo = 2000, using the Equation (1) suggested by Karabagias et al. [20]:

(1) $$C=\frac{A\times 10^{6}}{2000\times 100\times d}$$

where, *C* is the content of total carotenoids in mg/kg of oil and ‘*d*’ is the thickness of the spectrophotometer cell (1 cm).

### 2.5. Determination of Total Phenolic Content (TPC)

#### 2.5.1. Preparation of Extract for TPC

For the determination of TPC, there is a need to extract the total phenols from oil samples in methanolic solution to facilitate the analysis. The method used for the extraction of total phenols (from oil samples) was based on the procedure given by Fuentes et al. [21] and the TPC of the pumpkin extracts was analysed by utilising the Folin-Ciocalteu reagent method suggested by Singleton and Rossi [22]. For the extraction of phenolic compounds, 2.5 g of carotenoids extracts in corn oil were accurately weighed in a falcon tube (50 mL), and mixed with 5 mL of hexane by vortex (for two minutes). Further, to facilitate the extraction and phase separation, 3 mL of solvent mixture of methanol/water (60:40 *v*/*v*) was added and vortexed for 5 min. The methanol/water and hexane phases were separated after centrifugation (3500 rpm/10 min). The hexane phase was again treated or re-extracted with another 3 mL of solvent mixture (methanol/water, 60:40 *v*/*v*) for complete extraction of phenolics from hexane fraction. After centrifugation, both the layers were separated and pooled with their first extraction (methanol/water layer) samples (both layers, separately) and analysed for their total phenolic content, separately.

#### 2.5.2. Colourimetric Determination of Total Phenolic Content (TPC)

As mentioned earlier, the total phenolic content (TPC) of the above prepared extracts was analysed with the method given by Singleton and Rossi [22] and Dudonné et al. [23] after little modifications using gallic acid as standard. For the calculation of TPC, standard curve of gallic acid was plotted with varying concentrations from 10–100 µg/mL.

For standard curve, 400 µL of gallic acid (in different concentrations, 10–100 µg/mL) was mixed with 2 mL of Folin-Ciocalteu reagent (10 times diluted from original solution), followed by the addition of 1.6 mL of sodium carbonate solution (7.5%). For the determination of TPC of samples, same experiment was repeated as described above with treated, untreated corn oil, corn oil-carotenoids extracts and conventional extracts in place of gallic acid. After vortex mixing (for 1 min), the samples were kept for incubation (1 h) in dark at room temperature (25 ± 1 °C). After incubation period, the samples were analysed for their absorbance value at 765 nm. All samples were analysed in replicates (*n* = 9) and their results were expressed as mg Gallic acid equivalent (GAE)/g of the pumpkin extracts by calculating using Gallic acid standard curve (*R*= 0.9937).

### 2.6. DPPH Analysis

#### 2.6.1. Extraction Procedure for DPPH

For the DPPH analysis, the carotenoids extracts in corn oil were treated with methanol as per the method provided by Szydłowska-Czerniak et al. [24]. Briefly, 2 g of carotenoids extracts (in corn oil) were accurately weighted in 15 mL falcon tubes and mixed with 5 mL of methanol. These extracts were shaken for 30 min in the dark at room temperature and then kept for 30 min at −20 °C to facilitate the phase separation. The resultant extract (methanolic phase) was separated from oil and transferred into 50 mL falcon tubes. Remaining oil phase was re-extracted thrice as described above, and mixed (all three extractions). The methanolic extracts were stored in dark, in refrigerator (4 °C) and considered further for DPPH analysis.

#### 2.6.2. Determination of DPPH

The antioxidant capacity of carotenoids extracts was determined by DPPH assay using method previously given by Szydłowska-Czerniak et al. [24], after some modifications. For corn oil (carotenoids-extracts) sample analysis, 1.0 mL of above prepared methanolic extracts were added to 1.0 mL of methanol followed by the addition of 0.5 mL of DPPH* methanolic solution (12.0 mg/L). The solutions were then vortexed and kept in dark for 15 min. After incubation, the absorbance was recorded at 517 nm against pure methanol (blank) using spectrophotometer (SPECORD 250 Plus, Analytik Zena, Jena, Germany). The same procedure was followed for the conventional extracts and for Trolox standard (in the concentration ranges from 5.0 to 25.0 µg/mL). The radical scavenging activity was calculated using Equation (2):

(2) $$\%\;Inhibition=\frac{A_{control}-A_{sample}}{A_{control}}\times 100$$

where, *A_control_* = absorbance of DPPH radical + methanol; *A_sample_* = absorbance of DPPH radical + oil extracts (or standard solutions or conventional extracts (hexane/IPA)). The results were shown in % inhibition.

### 2.7. Colour Analysis

The colour analysis of the carotenoids extracts were evaluated using a X-Rite 964 spectrophotometer (RPimaging INC, Grand Rapids, MI, USA) by measuring L*, a* and b* values [25]. The X-Rite 964 242 spectrophotometer (RPimaging INC, Grand Rapids, MI, USA) has been used in several studies as standard reference [26,27,28,29] to analyse the colour of food samples as L*, a*, b* and ΔE* values. The L* refers the lightness, a* value refers the chroma on a green (–) to red (+) axis, b* value refers chroma on a blue (–) to yellow (+) axis. The colour difference between samples was calculated using Equation (3) [30]:

(3) $$\Delta E*=\;\sqrt{\Delta {L*}^{2}+\Delta {a*}^{2}+\Delta {b*}^{2}}$$

All measurements were taken in triplicate of individual triplicates (*n* = 9).

### 2.8. Oxidative Susceptibility by Rancimat Method

To analyse the effect of extraction methods i.e., UAE and MAE on the susceptibility to oxidation of the oils (used during extraction of carotenoids), the untreated oil (as control); treated (UAE and MAE) oil without carotenoids; and oil-extracts of carotenoids from pumpkin were analysed for oxidative stability by using Rancimat method [31]. For the determination of oxidative stability, 2.5 g of oil samples were accurately weighted into the reaction vessel of the Rancimat (892 Professional Rancimat, Metrohm, Switzerland) and placed in the heating sockets of the Rancimat. The temperature was set at 120 °C under a constant airflow rate at 20 L/h. During the oxidation process, the volatile acids formed due to oxidation of oil by means of processing air and temperature were recorded conductimetrically. The results were expressed as induction time (h) and were automatically printed by the instrument. The induction time is the time required by the conductivity curve to reach the inflection point that shows complete oxidation of oil samples. The induction time of treated, untreated corn oil and carotenoids-extract in corn oil were compared to check the antioxidant potential to preserve the shelf life of oils. The antioxidant potential of carotenoids extracts in corn oil obtained from pumpkin peels and pulp was evaluated using the protection factor (PF) by the method given by Nour et al. [32]. PF was calculated as the ratio between the induction period of the carotenoids extract in corn oil and the induction period of the control (treated with UAE/MAE without pumpkin samples). Regarding the PF values, if it is more than 1 (>1) then the carotenoids have antioxidant potential in the corn oil, and if the PF value is less than 1 (<1), it means carotenoids exhibit pro-oxidant behaviour. Higher the PF value, higher would be the antioxidant potential of the carotenoids in corn oil.

### 2.9. Statistical Analysis

All the experiments were carried out in replicates (*n* = 9) and the generated data were analysed using multivariate analysis of variance (M-ANOVA) followed by Duncan’s multiple range test and paired T- test (IBM SPSS^®^ Statistics v. 22.0, Armonk, NY, USA). A value of *p* < 0.05 was considered as statistically significant. Graphs were constructed by Microsoft Office Professional Plus 16 (Microsoft Co., Ltd., Redmond, WA, USA).

## 3. Results and Discussion

The peels and pulp of two varieties (codes: PM2 and PM4) of pumpkin were analysed for their physico-chemical (total carotenoids content, colour, and oxidative stability in oil) and antioxidant capacity (DPPH. Free radical assay and total phenolic content), using two innovative extraction techniques (UAE and MAE) synergised with green solvents and then compared with conventional extraction (CE) method. In the case of innovative techniques, we used ultrasound-assisted extraction and microwave-assisted extraction synergized with green solvents as corn oil. The study offered an efficient extraction technique, advantageous to utilising green solvents and promoting green consumerism.

### 3.1. Total Carotenoids Content (TCC)

The TCC was analysed from all the extracts in corn oil obtained from three different extractions methods and compared. Very interesting results obtained when extracts obtained from innovative or green extractions (MAE and UAE) were compared with conventional extractions. It was observed that the TCC of the pulp and peel extracts were almost two-fold in the case of innovative extractions as compared to conventional extractions in all the tested samples, as shown in Table 2.

**Figure 2:**
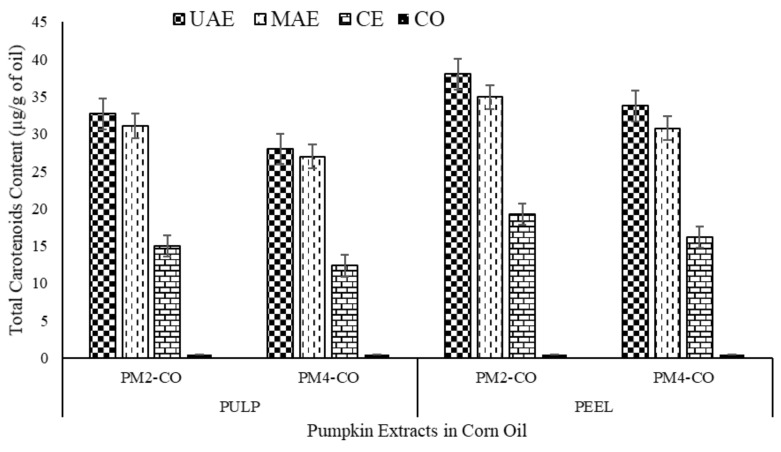
Effect of different extraction methods on the total carotenoids contents (µg/g of oil extracts) of pumpkin extracts in corn oil.

This may be due to the simulated effects of temperature and ultrasound or microwaves in innovative extractions, which played an important role in increasing the extraction efficiency of carotenoids from the pumpkin tissues. It was apparent that the extraction trend with respect to TCC was observed as: UAE > MAE > CE (conventional extraction); in terms of plant part i.e., peel > pulp; and in terms of PM2 > PM4 (in all samples). Results showed that the mean value of TCC of the pumpkin varieties PM2 and PM4 in both peels and pulp samples to vary significantly (*p* < 0.05) with different extraction methods (see Table 2 and Figure 2).

Highest TCC content was observed in the PM2 variety i.e., pulp had 32.69 ± 2.01 µg/g of oil extracts (326.9 µg/g of peel powder) and peel had 38.03 ± 4.21 µg/g of oil extracts (380.3 µg/g of peel powder) when treated with ultrasound-assisted extraction (UAE). Our results were corroborated with the results of De Carvalho et al. [33], who observed the TCC of landrace variety of pumpkin sample “A” to be 404.98 μg/g and that of sample “B” to be 234.21 μg/g. Similarly, Song et al. [34] reported a maximum yield of total carotenoids (trans- and cis- isomers) content to be 358 μg/g after extracted with UAE from peel of *Cucurbita moschata*. In addition, Salami et al. [35] compared the other innovative extractions and reported that the carotenoids content of *Cucurbita pepo* extract was found higher with subcritical water extraction (15.22 mg/100 g) when compared to supercritical fluid extraction (11.48 mg/100 g).

Further, in this study, PM2 variety was found to be significantly higher (*p* < 0.05) TCC in peel (38.03 ± 4.21 µg/g of oil extracts) as compared to PM4 variety (33.78 ± 1.76 µg/g of oil extracts), when extracted with ultrasound-assisted extraction (UAE). There was no significant difference observed on TCC of pumpkin pulp for both the varieties (PM2 and PM4) during both the extraction techniques (UAE and MAE extractions). On the other hand, the TCC of peels treated with UAE was significantly higher (PM2- 38.03 ± 4.21, PM4- 33.78 ± 1.76 µg/g of oil extracts) when compared with MAE treated samples (PM2- 34.94 ± 3.60, PM4- 30.78 ± 1.76 µg/g of oil extracts).

Contrarily, a previous study reported lower TCC from 22 cultivars of *Cucurbita moschata* ranged 7–138 µg/g [36]. Kandlakunta and co-workers reported the levels of total carotenoids about 2120 µg/100 g and β-carotene was about 1180 µg/100 g from *C*. *maxima* [37]. Li et al. reported that UAE synergised with sunflower oil as green solvent from fresh carrot, was found effective to extract the similar content of β-carotene about 334 mg/L after 20 min, whereas in the conventional extraction was about 321 mg/l after 60 min [17].

### 3.2. Antioxidant Study

#### 3.2.1. Total Phenolic Content (TPC)

**Figure 3:**
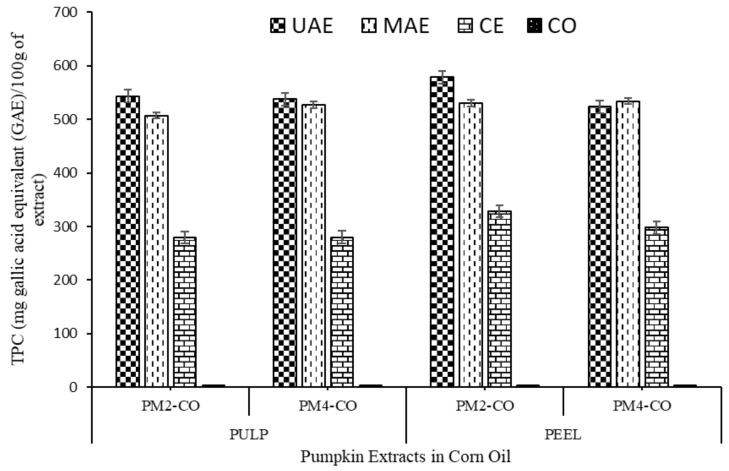
Effect of different extraction methods on total phenolic content (TPC) (mg gallic acid equivalent (GAE)/g of extract) of peel and pulp extracts of pumpkin in corn oil.

The TPC of the pumpkin peels and pulp extract represents the polyphenolic contents which are a major contributor to the antioxidant activity of pumpkin. TPC was analysed and expressed as mg GAE/100 g of extract (see Table 2 and Figure 3). TPC of pumpkin peels and pulp extracts were found significantly higher (*p* < 0.05) and ranged from 510.69 ± 5.50 to 588.68 ± 7.26 mg GAE/100 g of extract in the green extractions (UAE and MAE), than the conventional extracts (269.50 ± 2.17 to 318.46 ± 6.60 mg GAE/100 g of extract). It was observed that the innovative extraction methods employed (UAE and MAE with green solvent) in this study to extract carotenoids content were comparatively more effective than conventional extraction technique.

It was evident that ultrasound and microwaves can facilitate the disintegration of cells of plant tissues, which leads to the interruption of chemical bonds between macro and micro-molecules and eventually support the easy exits of phenolic compounds from the plant cells [38]. It was also observed that UAE method is more effective as compared to MAE technique in all of the tested samples, and this can be attributed to more penetrating power in the case of ultrasound waves as compared to microwaves. Moreover, microwaves increase the temperature during processing, which might be a cause of slight degradation of phenolic compounds.

Previously, Salami et al. [35] analysed the effect of subcritical water extraction (SWE) and supercritical fluid extraction (SFE) techniques on TPC of pumpkin (*Cucurbita pepo*) peels and reported that the TPC in SFE (353 mg GA/100 g) was higher as compared to SWE (213 mg GA/100 g). Similarly, TPC has been reported by Mala and Kurian [39] from pumpkin peels (5.21 mg GAE/g) and pulp (5.19 mg GAE/g). However, Altemimi et al. [40] observed lower TPC range between 38.46 to 43.85 mg/100 g in pumpkin (variety: *Libbys Select*) extracts when compared the conventional extraction with innovative extraction technique (UAE + conventional solvent- methanol).

#### 3.2.2. DPPH Antioxidant Activity

**Figure 4:**
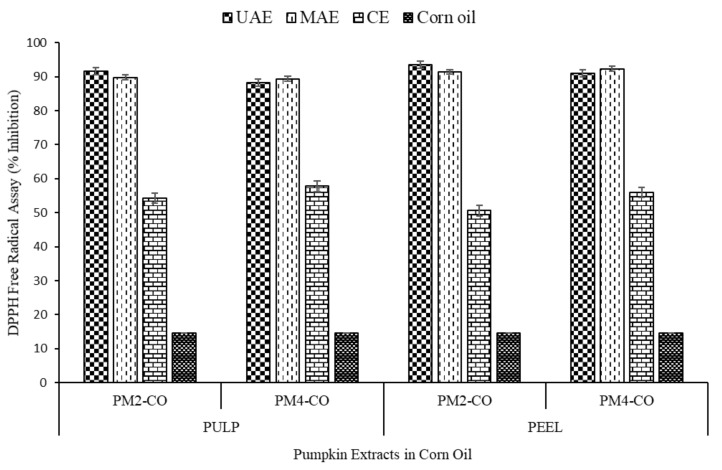
Effect of different extraction methods on the % inhibition of free radical by DPPH assay of pumpkin extracts in corn oil.

The pumpkin peels and pulp extracts were analysed for DPPH free radical assay, the results of which revealed that these extracts have good potential to scavenge free radicals. The results obtained have been depicted as percent inhibition in Table 2 and Figure 4.

The extracts obtained after extractions (UAE and MAE synergised with green solvent) showed promising free radical scavenging potential when compared to conventional extraction. The percent inhibition of DPPH was significantly higher (*p* < 0.05) or almost doubled in the case of extracts obtained from innovative extraction ranged from 88.32 ± 1.51 to 93.53 ± 0.30% than the conventional extracts ranged from 50.61 ± 1.44 to 57.79 ± 2.09% for all the tested samples. The higher differences in % inhibition of green and conventional extraction may be due to the synergistic effect of corn oil (14.51 ± 3.17%) present in green extraction samples, which itself is responsible to show some inhibition (%) towards DPPH free radical. Earlier, Altemimi et al. [40] observed similar DPPH radical scavenging antioxidant activity in pumpkin extracts (variety: Libbys Select) was ranged from 57.67 ± 0.48 to 64.85 ± 0.04% optimised via response surface methodology, when the conventional extraction was utilised in combination with innovative extraction technique (UAE + conventional extraction using methanol). Peel extracts of both of the varieties (PM2 and PM4) were observed with higher (but statistically insignificant) % inhibition as compared with their pulp extracts, using all the three extraction technologies (UAE, MAE and CE), as shown in Figure 4.

Moreover, there were non-significant differences observed in values of % inhibition from all the treated samples when comparing both the MAE and UAE extraction treatments. Extract of PM2 pumpkin-peel sample was recorded with highest (93.53 ± 0.30%) inhibition of DPPH free radicals after UAE extraction. Comparable to our results, Mala and Kurian [39] reported that the methanolic extracts of peel and pulp of pumpkin showed 80% inhibition at a concentration of 50 mg/mL. In contrast, lower values (37.04 ± 1.52%) of DPPH % inhibition in pumpkin peel extract is reported, which may be due to extraction technique, extraction solvent, variety of pumpkin and processing conditions [41]. The higher antioxidant capacity of pumpkin extracts in our study might be due to their enriched antioxidants level attributing to their hydrogen donating ability [42,43]. When comparing the antioxidant potentials of all the treatments, % inhibition trend was observed as PM2 > PM4, PEEL > PULP, UAE > MAE, UAE, and MAE > CE. There is not much literature representing green solvent extraction of pumpkin-carotenoids by using microwave-assisted extraction, even ultrasound-assisted extraction, especially with corn oil as green solvent.

### 3.3. Colour

The colour attributes i.e., L*, a*, b* and colour change (ΔE*) values of peel and pulp extracts of pumpkins are shown in Table 3. The peel and pulp extracts of both varieties i.e., PM2 and PM4 of pumpkin showed a wide range of colouring values for L* (1.37 ± 0.27 to 27.68 ± 1.64), a* (2.92 ± 0.61 to 22.24 ± 1.40), b* (2.16 ± 0.51 to 47.01 ± 2.57) and ΔE* (35.98 ± 1.56 to 63.67 ± 1.29). ΔE* value represents the colour change within the treatments and deals with more yellow or orange colour in the samples. The ΔE* values were found higher in both varieties: pulp (PM2 = 60.23 ± 0.08, PM4 = 63.87 ± 1.24) and peel (PM2 = 62.30 ± 0.55, PM4 = 63.67 ± 1.29) extracts obtained from ultrasound-assisted extraction. There was significant difference (*p* < 0.05) observed in all colouring attributes (L*, a*, b* and ΔE*) in all the extracts obtained from green extraction than the extracts obtained from conventional extraction.

There was significant difference (*p* < 0.05) observed in ΔE* values of pumpkin extracts prepared with UAE and MAE. Zhou et al. [44] analysed the colour attributes of three species of pumpkins i.e., *Cucurbita maxima*- L* = 38.08, a* = 3.30, b* = 17.60; *Cucurbita pepo*- L* = 32.67, a* = −0.13, b* = 1.68 and *Cucurbita moschata*- L* = 35.59, a* = 0.52, b* = 11.24. The variations in our results could be related with sample matrix where we used pumpkin extracts in corn oil and hexane/isopropyl alcohol (as the instrument is especially designed for solid or powdered samples); however, we calculated the colour parameters after subtracting the control sample readings from our treated samples. Similarly, Kulczyński et al. [9] carried out the colourimetric analysis of two varieties of pumpkin i.e., *Cucurbita moschata* and *Cucurbita pepo* and observed that the colouring parameters were in a wide range of L* (52.00–71.98), a* (−5.44–30.84) and b* (29.24–51.84). 

### 3.4. Determination of Susceptibility to Oxidation

The susceptibility to oxidation of oils containing carotenoids or control oil (treated with UAE or MAE without pumpkin samples with same processing conditions) was determined using the Rancimat method and expressed as induction period as represented in Table 4. The findings of the oxidative stability study showed that all the samples containing carotenoids from pumpkin peel and pulp exposed the values of protection factor (PF) ranging from 1.59 ± 0.01 to 1.81 ± 0.05, which recorded more than 1 (>1). From the results, it was concluded that all the carotenoids extracts obtained from pumpkin have antioxidant ability due to the presence of carotenoids, and as a result showed protecting effects on the oxidation of corn oil and enhanced the antioxidant stability of the medium.

There was a significant increase (*p* < 0.05) in the induction time of peel extracts (PF ranged from 1.71 ± 0.06 to 1.81 ± 0.05) as compared to pulp extracts (PF ranged from 1.59 ± 0.0 to 1.61 ± 0.00) in all carotenoids extracts in corn oil except for PM4 sample extracts obtained from MAE. There was no significant difference observed in the induction time of extracts treated with UAE and MAE, but MAE extracts showed lower values that may be due to decomposition of antioxidant potential of carotenoids in corn oil as a result of elevation in temperature during MAE-processing. In general, corn oil can be readily and quickly oxidised due to their rich content of polyunsaturated fatty acids [32], and hence the induction time was significantly higher in carotenoids extracted in corn oil as compared to the corn oil maintained as control. From the results, it can be concluded that peel and pulp of pumpkin have significant levels of carotenoids in corn oil extracts, which can prevent the oxidation of corn oil up to a significant time period. There is no previous literature available on pumpkin peel and pulp extracts in vegetable oils showing the induction period to enhance the antioxidant potential of vegetable oils.

However, in one of the studies reported earlier, the utilisation of dry tomato waste (5%) for extraction of tomato-carotenoids in vegetable oils and analysis of the effect of UAE, MAE and maceration on the induction time and oxidative susceptibility of the enriched oils were conducted [32]: it was reported that the induction time (protection factor) was much higher in MAE (1.35 ± 0.1) as compared to UAE (1.25 ± 0.0) in corn oil. This study also concluded that the use of various concentrations of tomato waste 2.5%, 5.0%, 10%, 20% for extraction, showed not much effect on the induction period (1.55 ± 0.1, 1.25 ± 0.0, 1.21 ± 0.1, 1.41 ± 0.1, respectively) of corn oil. It may be due to the elevation in temperature under high air flow during oxidation analysis, that the decomposition of carotenoids takes place, to some extent. Similarly, Benakmoum et al. [45] demonstrated that there was increase in induction time by 2.1 and 1.4 times when 30% of tomato puree and 10% of tomato peel were added in refined olive oil, respectively. These reports substantially support our observations in this study.

## 4. Conclusions

Combining the innovative technologies (UAE and MAE) with green solvent extraction to extract carotenoids from pumpkin peels and pulp could prove to be advantageous in terms of improving the extraction efficiency, reducing the extraction cost, time, energy and enhancing the yield of the target compound. The study offers a composite extraction technology to extract natural antioxidants from food wastes or by-products with a green-bio-refinery image. Corn oil is a well-recognised “green” alternative for petroleum-based or organic solvents and this can be safely used as an reliable extraction medium. This combination of innovative and green extraction technologies into a composite technology led the synergistic effect on the extraction process. UAE was observed with higher TCC, DPPH and TPC values as compared to MAE and conventional extraction. The apparent extraction trend observed for all the analyses (antioxidant and colouring) from different extraction techniques was UAE > MAE > CE. The extraction technology developed at laboratory-scale has high potential to be exploited further at industrial scale which can find wide applications in food, pharmaceutical and cosmeceutical-based industries. Besides this, the green solvent (corn oil) enriched with pumpkin-carotenoids can also be a good source of enrichment of antioxidant potential as well as enhance the aesthetic appeal of the food products in attractive consumer market.

As an initial approach, in this study we have focused on the comparison between conventional + solvent extraction with innovative + green solvent extraction (corn oil). However, future studies are required to study the extraction efficiency of other commonly studied green solvents such as ionic liquids or deep eutectic solvents and compare this with conventional solvent extractions. This would help to further confirm the effectivity of the selected green solvents.

## Figures and Tables

**Table 1:** Experiment design for the extraction of carotenoids from pumpkin varieties in corn oil using different extraction technologies.

Sample Names	Codes	UAE (Amplitude-20%, 30 Min)	MAE (130 W, 30 Min)	CE (Hex: IPA, 60:40 *v*/*v*, 60 Min)
*Cucurbita maxima* Var. Gold Nugget	PM2	Pulp-CO	Pulp-CO	Pulp
		Peel-CO	Peel-CO	Peel
*Cucurbita maxima* Var. Amoro F1	PM4	Pulp-CO	Pulp-CO	Pulp
		Peel-CO	Peel-CO	Peel

UAE: ultrasound-assisted extraction, MAE: microwave-assisted extraction, CE: conventional solvent extraction, and CO: corn oil.

**Table 2:** TCC (µg/g of oil extracts), total phenolic content (TPC) (mg gallic acid equivalent (GAE)/g of extract) and DPPH free radical assay (% inhibition) of peel and pulp extracts of pumpkin in corn oil (CO).

Sample Names	TCC (µg/g of Oil Extracts)		Total Phenolic Content (TPC) (mg Gallic Acid Equivalent (GAE)/g of Extract)		DPPH Free Radical Assay (% Inhibition)	
	Pulp	Peel	Pulp	Peel	Pulp	Peel
PM2-UAE-CO	32.69 ± 2.01 ^c,A^	38.03 ± 4.21 ^d,B^	555.20 ± 10.69 ^e,A^	588.68 ± 7.26 ^f,B^	91.55 ± 1.80 ^c,A^	93.53 ± 0.30 ^c,A^
PM2-MAE-CO	31.067 ± 2.45 ^c,A^	34.94 ± 3.60 ^c,d,A^	527.20 ± 5.69 ^d,A^	554.54 ± 10.25 ^e,B^	89.82 ± 1.36 ^c,A^	91.35 ± 0.94 ^c,A^
PM4-UAE-CO	28.01 ± 6.07 ^c,A^	33.78 ± 1.76 ^c,d,A^	524.48 ± 9.89 ^d,A^	547.94 ± 11.00 ^e,A^	88.32 ± 1.51 ^c,A^	90.90 ± 2.09 ^c,A^
PM4-MAE-CO	26.98 ± 6.12 ^c,A^	30.78 ± 2.78 ^c,A^	510.69 ± 5.50 ^c,A^	535.58 ± 3.84 ^d,B^	89.38 ± 4.51 ^c,A^	92.32 ± 1.43 ^c,A^
PM2-CE	15.01 ± 2.44 ^b,A^	19.21 ± 4.39 ^b,A^	269.50 ± 2.17 ^b,A^	318.46 ± 6.60 ^c,B^	54.29 ± 3.64 ^b,A^	57.79 ± 2.09 ^b,A^
PM4-CE	12.33 ± 1.90 ^b,A^	16.21 ± 2.52 ^b,A^	279.91 ± 4.53 ^b,A^	297.76 ± 2.14 ^b,B^	50.61 ± 1.44 ^b,A^	55.95 ± 4.62 ^b,A^
Corn oil	0.48 ± 0.007 ^a,A^	0.48 ± 0.02 ^a,A^	3.45 ± 0.21 ^a,A^	3.45 ± 0.21 ^a,A^	14.51 ± 3.17 ^a,A^	14.51 ± 3.17 ^a,A^

The values were given as mean ± SD (*n* = 9; triplicates of individual triplicates of pumpkin). The values followed by different superscripts (a–f) within the same column are significantly different (*p* < 0.05; Duncan’s multiple range test) from each other. Different letter superscripts (A,B) in each row indicate significant difference (*p* < 0.05).

**Table 3:** Colour attributes of pumpkin extracts produced from innovative and conventional extraction methods.

Sample Names	Colour Parameters							
	PULP				PEEL			
	L*	a*	b*	ΔE*	L*	a*	b*	ΔE*
PM2-UAE-CO	3.90 ± 0.33 ^a^	7.09 ± 0.71 ^b^	5.67 ± 0.21 ^a^	60.23 ± 0.08 ^c,A^	4.52 ± 2.26 ^b^	2.92 ± 0.61^a^	3.17 ± 1.43^a^	62.30 ± 0.55 ^c,B^
PM2-MAE-CO	1.84 ± 0.29 ^a^	13.85 ± 2.28^c^	3.78 ± 1.17 ^a^	43.65 ± 1.20 ^b,A^	1.37 ± 0.27 ^a^	7.47 ± 6.90 ^a^	2.16 ± 0.51^a^	45.08 ± 0.29 ^b,A^
PM4-UAE-CO	1.79 ± 0.56 ^a^	5.01 ± 1.49 ^a,b^	2.84 ± 0.99 ^a^	63.87 ± 1.24 ^d,A^	1.87 ± 0.59 ^a^	5.48 ± 1.98 ^a^	3.01 ± 1.03^a^	63.67 ± 1.29 ^c,A^
PM4-MAE-CO	1.43 ± 0.11 ^a^	3.76 ± 0.38 ^a^	2.50 ± 0.29 ^a^	44.55 ± 0.20 ^b,A^	1.54 ± 0.50 ^a^	4.15 ± 1.74 ^a^	2.41 ± 0.85^a^	44.56 ± 1.09 ^b,A^
PM2-CE	18.73 ± 2.57 ^b^	16.69 ± 1.16 ^d^	32.35 ± 3.78 ^b^	35.98 ± 1.56 ^a,A^	16.74 ± 1.08 ^c^	15.43 ± 3.08 ^b^	16.71 ± 8.71^b^	36.69 ± 1.34 ^a,A^
PM4-CE	19.89 ± 3.47 ^b^	19.59 ± 2.27 ^e^	34.02 ± 5.88 ^b^	36.81 ± 0.22 ^a,A^	27.68 ± 1.64 ^d^	22.24 ± 1.40 ^c^	47.01 ± 2.57^c^	38.49 ± 1.24 ^a,A^

L*—colour brightness; a*—colour in the range from green to red; b*—colour from blue to yellow. The values are given as mean ± SD (*n* = 9; triplicates of individual triplicates of pumpkin). The values followed by different superscripts (a–e) within the same column are significantly different (*p* < 0.05) from each other. Different letter superscripts (A,B) in each row indicate significant difference (*p* < 0.05).

**Table 4:** Rancimat analysis of carotenoids extracts of pulp and peels of pumpkin in corn oil.

Sample Names	Protection Factor (PF) of Carotenoids Extracts of Pumpkin in Corn Oil	
	Pulp	Peel
UAE_control	-	-
MAE_control	-	-
PM2-UAE-CO	1.61 ± 0.00 ^a,A^	1.81 ± 0.05 ^a,B^
PM2-MAE-CO	1.59 ± 0.02 ^a,A^	1.79 ± 0.05 ^a,B^
PM4-UAE-CO	1.59 ± 0.01 ^a,A^	1.74 ± 0.03 ^a,B^
PM4-MAE-CO	1.59 ± 0.01 ^a,A^	1.71 ± 0.06 ^a,A^

The values are given as mean ± SD (*n* = 9; triplicates of individual triplicates of pumpkin). The values followed by different superscripts (a) within the same column are not significantly different (*p* < 0.05) from each other. Different letter superscripts (A,B) in each row indicate there is significant difference (*p* < 0.05) in pulp and peels samples.

## Data Availability

Not applicable.
